# Testicular Dysfunction Ameliorative Effect of the Methanolic Roots Extracts of* Maytenus procumbens* and* Ozoroa paniculosa*

**DOI:** 10.1155/2017/8204816

**Published:** 2017-11-19

**Authors:** Nkosinathi David Cele, Nonhlakanipho Felicia Sangweni, Rebamang Anthony Mosa, Dambudzo Penduka, Geraldine Genevive Lazarus, Moganavelli Singh, Godfrey Elijah Zharare, Andy Rowland Opoku

**Affiliations:** ^1^Department of Biochemistry and Microbiology, University of Zululand, Private Bag X1001, KwaDlangezwa 3886, South Africa; ^2^School of Life Sciences, Biochemistry Department, University of KwaZulu-Natal, Durban 4000, South Africa; ^3^Department of Agriculture, University of Zululand, Private Bag X1001, KwaDlangezwa 3886, South Africa

## Abstract

The traditional use of medicinal plants in the management of sexual dysfunctions has a long history. This study investigated testicular dysfunction ameliorative effect of the methanolic roots extracts of* Maytenus procumbens *and* Ozoroa paniculosa *in a butanol-induced testicular dysfunction rat model. The rats in respective experimental groups were orally administered with the extract at 50 and 250 mg/kg bw, daily for 28 days. The cytotoxicity of the extracts was evaluated against HEK293, MCF-7, and HT29 cell lines. The extracts exhibited moderate (LC_50_ 30.3–330.2 *μ*g/mL) to weak (LC_50_ 200.8–438.4 *μ*g/mL) cytotoxicity level on the cancer and normal cells, respectively. While relatively lower serum testosterone levels and total sperm count along with decreased numbers of spermatogonia were noted in the untreated group, all these parameters were improved in the groups treated with the extracts at 250 mg/kg. Improved histomorphological changes of the testes were also observed when compared to the untreated group. While the extracts (at 250 mg/kg) increased serum reduced glutathione content and decreased malondialdehyde content, a relatively higher serum creatinine level was also observed in the treated animals group. The results indicate that the two plant extracts have potential to ameliorate testicular dysfunction.

## 1. Introduction

The maintenance of normal testicular function is crucial to male's health. Testicular dysfunction is the main underlying cause of male developmental and sexual dysfunctions. It may lead to a decline in fertility and androgen (testosterone) deficiency in males. Testosterone deficiency has debilitating effects (hypogonadism, infertility, low libido, poor muscle strength, and erectile dysfunction) in the quality of life of men [1, 2]. Various factors, either external or internal, are known to compromise testicular functions. Such factors include exposure to toxic substances, diabetes mellitus, oxidative stress, and aging [3, 4]. The testicles are also vulnerable to oxidative stress due to their higher level of polyunsaturated fatty acids (PUFA) and presence of potential reactive oxygen species (ROS) generating system [5, 6].

Despite an increasing number of men's health clinics worldwide and the use of pharmaceutical agents to manage complications of testicular dysfunction, substantial number of men, particularly living in rural areas, continue to consult traditional healers to improve their sexual functioning [7]. The use of medicinal plants, by traditional healers against sexual dysfunctions, has a long and extensive history [8–10]. Ethnopharmacological survey of traditional healers in Northern Kwa-Zulu Natal (South Africa) indicates that powdered roots of* Maytenus procumbens *and* Ozoroa paniculosa* are commonly used by traditional healers in the management of testicular dysfunctions (Personal communication).

*Maytenus procumbens* (L.F.) Loes (Celastraceae), commonly known as Dune Koko (English) and* Umakhweni* (Zulu), is a densely bush plant (about 6 m in height) native to South Africa and predominant in the KwaZulu-Natal (KZN) province. It is also found in South America, North Africa, and East Asia [11]. The leaf extract of* M. procumbens* has been reported to possess anticancer activity [11].* Ozoroa paniculosa* (Sond.) R & A Femandes, (Anacardiaceae), commonly known as resin tree (English) and* Umfico *(Zulu), is an evergreen, semideciduous, small to medium sized single stemmed tree (up to 9 m). The powdered stem bark of* O. paniculosa* is used by Zulu traditional healers to treat acute inflammatory condition of the chest [12]. This study is aimed at evaluating effect of methanolic roots extracts of* M. procumbens* and* O. paniculosa* against testicular dysfunction in male rats. Effect of the extracts on fertility is not covered in the current study.

## 2. Materials and Methods 

### 2.1. Plant Material Preparation and Extraction

Fresh roots of* M. procumbens *and* O. paniculosa* were collected from Ndumo and Mhlabayalingana, KZN, South Africa, in collaboration with a local traditional healer. The botanical identification of the plants was confirmed by Dr. N.R Ntuli and Dr. T.H.C Mostert at the Botany Department, Zululand University. The plants materials were air-dried and ground to powder. Methanol was used to separately extract (1 : 5 w/v; 48 h on a shaker at 150 rpm; room temperature) the powdered plants materials. The filtrates were concentrated in* vacuo *(at 40°C). The obtained methanol-free crude extracts were safely stored at 4°C until use. Fresh working solutions were prepared by reconstituting the crude extract in distilled water just before use.

### 2.2. Phytochemical Screening

Phytochemical analysis (Qualitative) of the plants material was performed following the standard procedures described by Odebiyi and Sofowora [13]. The plants' materials were screened for basic phytochemicals such as tannins, alkaloids, saponins, steroids, flavonoids, and terpernoids.

#### 2.2.1. Total Phenolic Content (TPC)

The phenolic content of the crude extracts was colourimetrically quantified following the Folin-Ciocalteu reagent method [14]. Crude extract (0.5 mg/mL) was well mixed with 1.5 mL of Folin-Ciocalteu reagent and 7.5% v/w sodium carbonate (1.2 mL). Following 30 mins incubation, absorbance was read at 765 nm. The total phenolic content of the plant extract was determined as gallic acid equivalent from a gallic acid calibration curve and expressed as mg/g dry plant material.

#### 2.2.2. Flavonoid Content (FC)

The method described by Ordońez et al. [15] was followed to colourimetrically determine the FC of the extracts. Crude extract (0.5 mg/mL) and 0.5 ml of alcoholic aluminium chloride (2%) were well mixed together. After incubation (1 h at 25°C), absorbance was read at 420 nm. The total flavonoid content of the extract was determined from a quercetin calibration curve and expressed as mg/g dry plant material.

### 2.3. Cytotoxicity Assay

The cytotoxic effect of the extracts was tested against normal human embryonic kidney (HEK293), human colorectal adenocarcinoma (HT29), and breast cancer (MCF-7) cell lines, using MTT assay [16]. The cells were all obtained from the American Tissue Culture Collection (ATCC). The proliferating cells (1.8 × 10^4^ cells/mL) were exposed to the extract for 48 h at 37°C. The cells viability was tested with the tetrazolium salt and after 4 h incubation at 37°C, the formed formazan crystals were solubilised with dimethyl sulfoxide (DMSO). The optical density of the solutions was measured at 570 nm using a Mindray-96A microplate reader. Percentage inhibition of cell viability was calculated using the formula

(1) $$\begin{array}{c} \%\;cell\;death=\left[\;\frac{\left(Ac-At\right)}{Ac}\times 100\;\right], \end{array}$$

where Ac is the absorbance of control and At is the absorbance in the presence of extract.

### 2.4. Effect of the Extract on Testicular Dysfunction in Rats

Approval for use of laboratory science animals and procedures was issued by the University of Zululand Research Ethics Committee (UZREC 171110-030 PGM 2015/198). Sprague-Dawley rats (150–200 g) of either sex were obtained from the animal unit of the Biochemistry Department, University of Zululand. Rats were kept under standard laboratory conditions (at 23 ± 2°C, 50 ± 5% humidity, and day-night cycle). The animals had access to fresh food and drinking water at leisure for the duration of the experiment. The rats were separated according to gender (maximum of four rats per cage) and were allowed to acclimatize for at least five days before the experiment commenced.

The effect of the extracts on testicular dysfunction was evaluated using the butanol-induced testicular dysfunction in rat model [17], with some modifications. The sexually active male Sprague-Dawley rats received an intraperitoneal injection of* n*-butanol at 25 mg/kg body weight for four days at two-day intervals to induce testicular dysfunction. Four days after the induction of testicular dysfunction, the animals were mated with estrous female rats to establish their baseline sexual performance. The animals were then randomly divided into seven groups (A–G) of five rats per group and put on their respective treatments as shown as follows:

  Group A: normal control group, received water (a carrier solvent).
  Group B: testicular dysfunction induced (untreated group), received water only.
  Group C: testicular dysfunction induced, received sildenafil at 20 mg/kg.
  Group D: testicular dysfunction induced, received 50 mg/kg* M. procumbens*.
  Group E: testicular dysfunction induced, received 250 mg/kg* M. procumbens*.
  Group F: testicular dysfunction induced, received 50 mg/kg* O. paniculosa*.
  Group G: testicular dysfunction induced, received 250 mg/kg* O. paniculosa*.

All the animals were administered (oral dose) with their respective drugs daily for 28 days. A group of female rats (150–200 g) received two consecutive subcutaneous injections of progesterone (7.5 mg/kg bw), at 48 h intervals for 96 h to induce estrous before being introduced to male rats. To assess the rats' sexual behavior, each male rat from various treatment groups was individually subjected to one estrous female rat in a separate cage for 30 min. The number of mounts were recorded and compared between the groups. Body weight changes of the male rats were recorded for 28 days at seven days intervals. At the end of the experimental period, the male rats were euthanised under anaesthesia, blood was immediately collected by cardiac puncture, and the testes were removed and weighed. The collected tissue samples were used for biochemical parameters and histological analysis, respectively.

### 2.5. Biochemical Estimation of Antioxidant Status, Testosterone, Creatinine, and Liver Function Enzymes

Serum levels of testosterone, creatinine, aspartate aminotransferase (AST), and alanine aminotransferase (ALT) were determined using standard laboratory procedures (Global Laboratory & Viral Laboratory, Richards Bay). The serum glutathione (GSH) and malondialdehyde (MDA) contents as well as activities of antioxidant enzymes (catalase, CAT; superoxide dismutase, SOD) were estimated using respective commercial activity assay kits (Sigma-Aldrich), suitable for rat samples, following manufacturers' instructions.

### 2.6. Sperm Count

Epididymides were rinsed in ice-cold 0.01 M phosphate buffer (pH 7.4) and kept at −80°C until used. Sperm count was estimated following the method described by D'Souza [18] and Adeiza and Minka [19], with some modifications. The epididymides (0.2–0.3 g) were thawed and minced in 1 ml of 0.1 M phosphate buffered saline (pH 7.2). The suspension was filtered and the filtrate was mixed (10 : 1) with 100 *μ*L of 1% aqueous nigrosine-eosin Y. The mixture was incubated for 30 min at room temperature to allow staining of the sperm cells. This was followed by placing the sperm suspension (10 *μ*L) on the microscope slides (1 cm^2^) for sperm counting. The sperm count was performed as per standard procedure using an optical microscope with 400× magnification. The total number of sperm cells was calculated from the following formula:

(2) $$\begin{array}{c} S=C\times V\times CF, \end{array}$$

where *C* is number of counted spermatozoa; *S* is Sum total per animal; *V* is dilution (10^4^); and *CF* is factor of the camera (1.25).

### 2.7. Histological Analysis

Testicles were removed, weighed, and stored in 40% formalin. The tissues were processed routinely and stained with haematoxylin and eosin. Histopathological analysis was carried out by a qualified pathologist (Vetdiagnostix Laboratories, Pietermaritzburg) with no prior knowledge of the animal groups and their treatment.

### 2.8. Data Analysis

Unless stated otherwise, the data were presented as mean ± SEM. The results were analyzed with one-way ANOVA followed by Dunnett's tests using GraphPad Prism (v6.01). The differences were statistically significant where *p* < 0.05. The LC_50_ values were determined using the Cheburator version 1.2.0 software.

## 3. Results

### 3.1. Phytochemical Screening

The phytochemical screening of the roots of* M. procumbens *and* O. paniculosa* showed the presence of most phytoconstituents that were screened for (Table 1). It is noted that even though flavonoids and steroids were not detected in* M. procumbens*, the roots of the plant showed high terpenoid content. Despite the lower TPC (0.063 mg/g) in* O. paniculosa*, a relatively higher FC (0.102 mg/g) in the roots of the plant was recorded.

### 3.2. Cytotoxicity

The cytotoxic effect of the extracts on the tested cell lines varied from moderate to weak toxicity (Table 2). Both* M. procumbens* and* O. paniculosa* extracts showed lower lethality (LC_50_) values of 80.0 and 30.3 *μ*g/mL, respectively, on HT29 cells.

### 3.3. Biochemical Analysis of the Serum Levels of Liver Function Enzymes and Creatinine

Table 3 shows results of the effect of the extracts on serum levels of liver function enzymes (AST and ALT) and creatinine. Relatively lower levels of ALT and AST along with increased levels of creatinine were observed in the groups that received the extracts when compared to the untreated group. A similar effect to the extract-treated groups was also observed in the sildenafil treated group.

### 3.4. Biochemical Analysis of the Serum Antioxidant Status

The results of the effect of the extracts on the antioxidant status are presented in Table 4. Treatment of the animals with the extracts proved to increase and boost the animals' antioxidant status. This was evidenced by a general increase in GSH content (for both extracts at 250 mg/kg) with a significant (*p* < 0.05) decrease in serum MDA level, a product of lipid peroxidation.

### 3.5. Effect of the Extracts on Testicular Weight, Serum Testosterone Levels, and Total Sperm Count

The effect of the extracts on testicular weight, serum levels of testosterone, and total sperm count was evaluated and the results are given in Table 5. Relatively lower testicular weight, testosterone levels, total sperm count, mounting frequency, and smaller body weight changes were observed in untreated control group. However, increase in the tested parameters was evident in animals treated with the extracts at a higher concentration of 250 mg/kg. A marked increase in testicular weight, serum testosterone, and total sperm count in the animals treated with* M. procumbens* (250 mg/kg) correlated with an observed significant (*p* < 0.001) increase in the animals' mounting frequency. All the measured parameters were also improved in the sildenafil treated group.

### 3.6. Histopathological Analysis of the Testes

**Figure 1:**
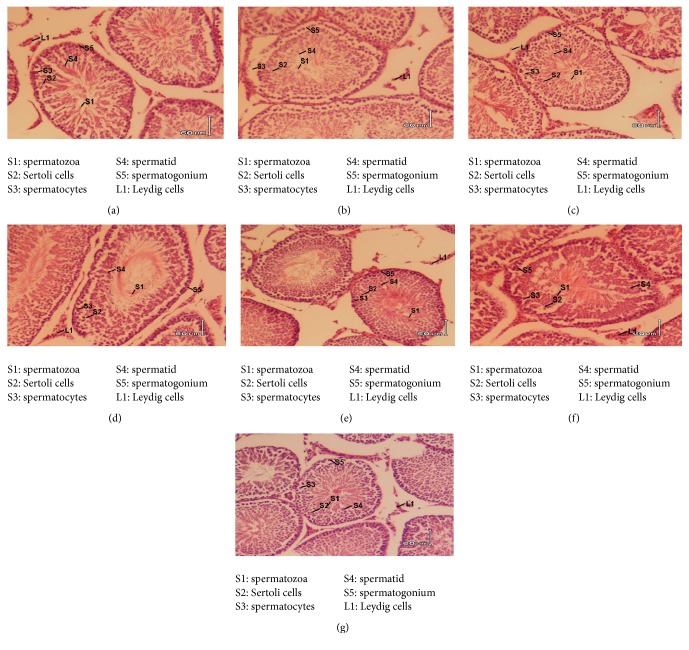
Histological analysis of the testes. All specimens were prepared as 60 *μ*m thick sections stained with haematoxylin and eosin. (a) Testis' cross section from normal control; (b) section from untreated testicular dysfunctional rats; (c) testicular dysfunctional rats treated with sildenafil (20 mg/kg); (d) testicular dysfunctional rats treated with* M. procumbens* (50 mg/kg); (e) testicular dysfunctional rats treated with* M. procumbens* (250 mg/kg); (f) testicular dysfunctional rats treated with* O. paniculosa* (50 mg/kg); (g) testicular dysfunctional rats treated with* O. paniculosa* (250 mg/kg). Magnification: ×200.

Figure 1 shows histopathological analysis of the testes following the 28 days treatment period of the animals with the extracts. While a complete differentiation of the seminiferous tubules with regular arrangement of cells was observed in the normal control, various morphological changes were evident in the tissue of the untreated group. In addition to the degeneration of the basal layer of the epithelium (germinal) which resulted to decreased amount of spermatogonia, loose and irregular arrangement of the cells in the tubules was observed in the untreated group (Figure 1(b)). This was also accompanied by a considerable loss of some cellular material from the seminiferous epithelium. However, the histomorphological changes brought about by the treatment of the animals with the extracts (at 250 mg/kg) were comparable to those of the normal control group animals. A densely packed spermatogonia and Sertoli cells (that rested on the basement membrane) were observed following the animals' treatment with the extracts. These cells seemed to be normally surrounded by myofibroblast layer (Figures 1(e) and 1(g)).

## 4. Discussion

Testicular dysfunction, due to either endogenous or exogenous factors, may result in a decline in male sexual activeness, androgen synthesis, and fertility. An increasing body of evidence supports the use of medicinal plants, parts or whole plant, in the management of testicular dysfunctions [8–10]. In this study, effect of the methanolic roots extracts of* M. procumbens *and* O. paniculosa *in butanol-induced testicular dysfunction in male rats is reported. Alcohol intoxication is linked to decreased serum levels of testosterone, loss of spermatogenic activity, and testicular atrophy [20, 21]. Testosterone synthesis is key to the development of male secondary sexual characteristics and enhancement of sexual desire and also in sperms production [22]. The observed increase in sperm count and higher levels of serum testosterone in the treated groups (Table 5) support the extracts' potential steroidogenic and spermatogenic activity.

The testicular atrophy is primarily associated with shrinkage of the seminiferous tubules and loss of sperm cells [23]. The observed increase in the testicular weight and regeneration of spermatogonia in the seminiferous tubules of the rats treated with the extracts (Figures 1(e) and 1(g)) further support the spermatogenic of activity of the extracts. Treatment of the animals with the extracts (at 250 mg/kg) clearly improved the histomorphological changes of the testes when compared to the untreated group. The ameliorative effect of the extracts could be attributed to the presence of phytoconstituents such as saponins, flavonoids, and alkaloids (Table 1) which are commonly implicated in the restoration of testicular function [24, 25].

Furthermore, therapeutic effect of a number of medicinal plants against testicular dysfunction has also been associated with their antioxidant activities [10]. The high content of polyunsaturated fatty acids and the presence of potential ROS system in the testes render them highly vulnerable to oxidative stress [5, 6]. The increased ROS level in seminal plasma may cause disruption of spermatogenesis and damage to sperm cells [26, 27]. Thus, the presence of phenolic compounds (Table 1), which are well known of their antioxidant activity [28], in the extracts could prove vital in their protection against testicular injury. The increased GSH content and thus inhibition of lipid peroxidation (Table 4) in the groups administered with the extracts (at 250 mg/kg) support the antioxidant protection offered by the extracts.

Despite their efficacy, evaluation of the cytotoxic effects of plants used by traditional healers for medicinal purposes is also crucial. The use of both normal and cancer cells in this study was solely to evaluate general cytotoxic effect of the extracts. Toxicity of crude extracts is categorized into highly toxic (LC_50_ ≤ 20 *μ*g/mL), moderately toxic (LC_50_ = 21–200 *μ*g/mL), or weakly toxic (LC_50_ = 201–500 *μ*g/mL) [29]. The results obtained (Table 2) indicate weak toxicity effect of the extracts on the normal cells (HEK293). The weak toxic effect of* M. procumbens* extract on normal cells is consistent with the report of Ahmed et al. [30]. Therapeutic agents with selective effect between normal and cancer cells are highly demanded. The relatively higher toxicity levels of the extracts on the cancer cells, particularly HT29 cells, than normal cells (HEK293) indicate their selectivity. Thus these extracts could also be potentially explored in cancer therapy research. Furthermore, the noted elevated serum creatinine levels, a common diagnostic biomarker of renal dysfunction, in the groups treated with the extracts (Table 3) cannot be overlooked. This could be an indication of a dose-dependent potential renal threat of the extracts.

## 5. Conclusion

It was concluded that the methanolic root extracts of* M. procumbens *and* O. paniculosa *have properties to potentially ameliorate testicular dysfunction. The results from this study support the use of the roots of these plants in folk medicine to manage testicular dysfunction and its related complications. While the extracts exhibited potential efficacy against testicular dysfunction, the observed potential cytotoxicity and/or renal toxicity of the extracts suggest that the plants be medicinally used with caution. Evaluation of erectogenic effect of the extracts and isolation of their active compound(s) are recommended for further work.

## Figures and Tables

**Table 1:** Phytochemical analysis of *M. procumbens *and* O. paniculosa *roots.

Phytochemical	*M. procumbens*	*O. paniculosa*
Terpenoids	++	+
Saponins	++	+
Flavonoids	−	++
Alkaloids	+	−
Steroids	−	+
Tannins	++	−
Anthraquinones	++	+
cardiac glycosides	++	++
TPC (mg/g)	0.083 ± 0.004	0.063 ± 0.001
FC (mg/g)	0.00 ± 0.000	0.102 ± 0.003

++ indicated high concentration was recorded if a definite heavy precipitate observed. + indicates low concentration was recorded if the reagent produces only slight opaqueness. – indicates not detected.

**Table 2:** LC_50_ (*µ*g/ml) values of *M. procumbens* and *O. paniculosa* against HEK, MCF-7, and HT29 cells.

Extracts	HEK 293 cells	MCF-7 cells	HT29 cells
*M. procumbens*	438.4 ± 38.4	330.2 ± 21.2	80.0 ± 28.9
*O. paniculosa*	356.1 ± 14.4	230.3 ± 27.9	30.3 ± 7.5

Data were expressed as mean ± SD (*n* = 3).

**Table 3:** Effect ofthe extracts on serum levels of creatinine, AST, and ALT.

Group	Creatinine (*μ*moles/L)	AST (IU/L)	ALT (IU/L)
Normal control	16 ± 2.58	164 ± 8.04	71 ± 6.39
Untreated	16 ± 2.95	181 ± 12.02	83 ± 2.79
Sildenafil (20 mg/kg)	30 ± 2.34	149 ± 14.84	76 ± 7.60
*O.p *(50 mg/kg)	21 ± 0.47	175 ± 19.32	62 ± 1.65
*O.p *(250 mg/kg)	27 ± 0.70	136 ± 5.42^*∗*^	54 ± 0.70^*∗*^
*M.p *(50 mg/kg)	21 ± 1.25	150 ± 23.85	72 ± 1.73
*M.p *(250 mg/kg)	34 ± 2.31^*∗*^	155 ± 3.90	58 ± 3.59

Data were expressed as mean ± SD, *n* = 5. ^*∗*^*p* < 0.05 versus untreated group.

**Table 4:** Effect of the extracts on serum levels of SOD, CAT, GSH, and MDA in the testicular dysfunction induced rats.

Group	SOD (units/mL)	CAT (*μ*moles/min/mL)	GSH (nmoles/*μ*L)	MDA (nmoles/mL)
Normal control	18.9 ± 0.08	6.13 ± 0.02^*∗*^	7.3 ± 0.04	0.08 ± 0.01
Untreated	16.2 ± 0.02	3.37 ± 0.00	4.4 ± 0.01	0.16 ± 0.02
Sildenafil (20 mg/kg)	13.3 ± 0.01	3.33 ± 0.00	4.9 ± 0.03	0.14 ± 0.01
*O.p *(50 mg/kg)	12.8 ± 0.00	3.36 ± 0.09	6.8 ± 0.01	0.17 ± 0.04
*O.p *(250 mg/kg)	9.6 ± 0.00	3.01 ± 0.04	9.8 ± 0.02^*∗*^	0.04 ± 0.01^*∗*^
*M.p *(50 mg/kg)	10.6 ± 0.00	3.73 ± 0.00	3.2 ± 0.06	0.06 ± 0.02
*M.p *(250 mg/kg)	11.1 ± 0.07	4.27 ± 0.05	5.4 ± 0.01	0.03 ± 0.00^*∗*^

Data were expressed as mean ± SEM, *n* = 5. ^*∗*^*p* < 0.05 versus untreated group.

**Table 5:** Effects of plant extracts on body weight, testicular weight, serum testosterone levels, total sperm count, and mounting frequency.

Group	BWC (g)	TW (g)	Testosterone (nmol/L)	TSC (10^4^)	MF (per 30 min)
Normal control	49 ± 16.26	3.40 ± 0.48	6.99 ± 3.09^*∗*^	47 ± 05.81	19 ± 1.02^*∗*^
Untreated	39 ± 16.23	2.76 ± 0.08	3.01 ± 2.82	35 ± 07.94	09 ± 1.00
Sildenafil (20 mg/kg)	50 ± 15.44	3.55 ± 0.41	14.93 ± 8.34^*∗∗*^	37 ± 05.92	64 ± 1.01^*∗∗*^
*O.p* (50 mg/kg)	65 ± 17.47	3.33 ± 0.20	2.12 ± 1.54	53 ± 20.17	16 ± 3.09
*O.p* (250 mg/kg)	97 ± 12.26^*∗*^	3.62 ± 0.62	6.60 ± 3.51^*∗*^	74 ± 10.56^*∗*^	18 ± 3.54^*∗*^
*M.p* (50 mg/kg)	44 ± 11.60	4.21 ± 0.41	2.58 ± 0.71	46 ± 2.56	32 ± 3.11^*∗∗*^
*M.p* (250 mg/kg)	48 ± 13.23	4.29 ± 0.11	10.95 ± 3.42^*∗*^	49 ± 14.13	46 ± 1.20^*∗∗*^

BWC: body weight change; TW: testicular weight; TSC: total sperm count; MF: mount frequency; *M.p*:* Maytenus procumbens; O.p*:* Ozoroa paniculosa. *Data were expressed as mean ± SEM. Data were expressed as mean ± SEM, *n* = 5. ^*∗*^*p* < 0.05 and ^*∗∗*^*p* < 0.001 versus untreated group.
